# Supplementary material for: Osmolarity-independent electrical cues guide rapid response to injury in zebrafish epidermis
Source: eLife. 2020 Nov 23;9:e62386. doi: 10.7554/eLife.62386 (PMC7721437; doi:10.7554/eLife.62386)
Supplement: Supplementary file 1. [file elife-62386-supp1.docx]

Primers used in Kennard and Theriot - Osmolarity-independent electrical cues guide rapid response to injury in zebrafish epidermis

Note that hyphens have been added for readability but are not part of the sequence.

If uppercase and lowercase appear in the same primer, UPPERCASE indicates the addition of non-homologous sequence, such as attB sites added to the primers for Gateway recombination. Lowercase indicates regions of homology with the template.

| Primer ID | Sequence (5’ to 3’) | Notes |
| --- | --- | --- |
| 1 | GGGGACAAGTTTGTACAAAAAAGCAGGCTTAGCCACCATGGGTTCTCATCATCATC | Forward primer used to amplify GCaMP6f-P2A-nls-dTomato from template. Underlined sequence introduces a Kozak sequence prior to the translation start site |
| 2 | GGGGACCACTTTGTACAAGAAAGCTGGGTTGCCGTCGACTTACTTGTACAGC | Forward primer used to amplify GCaMP6f-P2A-nls-dTomato from template. |
| 3 | GGGGACAAGTTTGTACAAAAAAGCAGGCTGGatggtgagcaagggcgaggag | Forward primer used to amplify mNeon green from template, beginning at the start codon. |
| 4 | GGGGACCACTTTGTACAAGAAAGCTGGGTCcttgtacagctcgtccatgc | Reverse primer used to amplify mNeonGreen from template, not including the stop codon. |
| 5 | GGGGACAGCTTTCTTGTACAAAGTGGTAatggtgtctaagggcgaagag | Forward primer used to amplify mRuby3 from template, beginning at the start codon |
| 6 | GGGGACAACTTTGTATAATAAAGTTGTttacttgtacagctcgtccatgcc | Reverse primer used to amplify mRuby3 from template, including the stop codon |
| 7 | CAGGCTGGAGACGTGGAGGAGAACCCTGGACCTgacccagctttcttgtac | Primer used for Q5 mutagenesis to insert a Gly-Ser-Gly linker to the C-terminus of mNeonGreen |
| 8 | CTTCAGCAGGCTGAAGTTAGTAGCTCCGCTTCCcttgtacagctcgtccatg | Primer used for Q5 mutagenesis to add a P2A site to the C-terminus of mNeonGreen (after the Gly-Ser-Gly linker) |
| 9 | TGAGAGTGGCCCCGGCTGCATGAGCTGCAAGTGTGTGCTCTCCtaaacaactttattatacaaagttgg | Primer used for Q5 mutagenesis to add a GGSGGGSGG linker to the C-terminal end of mRuby3 |
| 10 | TCAGGAGGGTTCAGCTTGCCGCCGCTGCCGCCGCCGCTGCCGCCcttgtacagctcgtccatg | Primer used for Q5 mutagenesis to add a CAAX tag to the C-terminal end of mRuby3 |
